# Entrepreneurial Resilience: A Case Study on University Students

**DOI:** 10.3390/ijerph19052589

**Published:** 2022-02-23

**Authors:** Elisabet Montoro-Fernández, Antonio Ramón Cárdenas-Gutiérrez, Antonio Bernal-Guerrero

**Affiliations:** 1 Department of Communication and Education, Faculty of Social and Human Sciences, University Loyola Andalucía, 41704 Seville, Spain; 2 Department of Theory and History of Education and Social Pedagogy, Faculty of Education Sciences, Seville University, 41013 Seville, Spain; acardenas1@us.es (A.R.C.-G.); abernal@us.es (A.B.-G.)

**Keywords:** resilience, entrepreneurship, salutogenesis, university students, sense of coherence, general resilience resources, case study, mixed research

## Abstract

Entrepreneurial resilience refers to the capacity to face, overcome and project oneself after suffering life events with a negative impact. Emerging adulthood and the characteristics of university life facilitate the occurrence of stressful situations that can affect well-being. The aim of this phenomenological research is to explore the strategic components of entrepreneurial resilience and how young university students have shaped their entrepreneurial resilience after experiencing negative life events. The present research is a multiple case study that was developed through a mixed methodology. The methodological sequence was quantitative and qualitative, with priority given to the qualitative phase of the research. Ten university students with high levels of resilience were interviewed. The data were analysed using thematic analysis. The results indicate that resilience is built through intrapersonal and exopersonal processes. These processes make up a set of strategic dimensions related to entrepreneurial behaviour that are used for the construction of personal projects.

## 1. Introduction

Psychological resilience is a conceptually complex term that raises discrepancies in its understanding depending on the personality traits, processes and outcomes involved [1,2,3]. This is corroborated by bibliometric studies that show a broad, diverse and fragmented state of the art [4,5]. Despite this conceptual heterogeneity, certain recurrent terms related to resilience have been extracted, such as coping capacity, adversity, risk factors, stressful situations, suffering and positive adaptation [6]. This terminological list leads to an approach of resilience as a multidimensional construct that involves the combination of biological, social, cultural and psychological processes [7] promoting adaptation and resistance to circumstances that are detrimental to physical and psychological well-being, whether in the social, personal, family or work sphere [8,9,10].

There is a widely accepted view of entrepreneurship as being directly related to business and understood as the process of initiation, creation, development and management of business projects [11,12,13,14]. However, in the educational context, entrepreneurship has two different notions: “enterprise education” and “entrepreneurship education” [15]. The concept of “enterprise education” is related to the development of generic skills linked to the projection of life in an infinite number of situations [16]. On the other hand, the term “entrepreneurship education” is linked to entrepreneurial creation and business [17]. Given this distinction, we adopt the construct “enterprise education”, as it represents the adoption of a more global approach centred on the capacity to initiate, transform, adapt, act and develop in the face of the opportunities or difficulties that affect people in their life cycle [18]. In line with the previous research [19,20,21], we associate “Enterprise education” with personal initiative, i.e., a set of self-initiated, proactive and sustainable actions that are sustainable over time and have the capacity to transform the context in which the subject finds him/herself [22].

The intersection of entrepreneurship and resilience has been studied from the theories of business organisation, either as a capability [23], process [24], operation [25] or result [26]. Nonetheless, all these perspectives constitute an interpretation of entrepreneurial resilience limited exclusively to the workplace. However, from a more holistic perspective, and related to personal development and growth, the term entrepreneurial resilience is reinterpreted in this paper, detaching it from the exclusive interpretation of the theories of business organisation and conceptualising it from the broader construction of personal identity, understood as a dynamic, evolutionary and social process, in which entrepreneurial initiative is included. The shaping of identity involves choosing, initiating and carrying out life activities that are part of the personal experience. This approach converges with lines of research focused on goal-oriented human behaviour, of a profuse projection, such as personal projects [27], personal aspirations [28], personal tasks [29] or possible selves [30]. All of them are linked to the narrative construction of personal identity, incorporating not only the vital charge of past and present experiences, but also the direction and management of expectations [31]. In this way, we conceptualise entrepreneurial resilience not only as the ability to face and manage life’s adversities efficiently and effectively (resilience), but also to project the self into the future, through the implementation of strategies related to entrepreneurial orientation and behaviour [32,33]. The incorporation of entrepreneurial behaviour into resilience processes promotes the configuration of personal projects, aspirations and areas, where negative episodes in life are adequately integrated (entrepreneurship), configuring a congruent biographical narrative.

Entrepreneurial resilience is framed within the salutogenic model. This theory focuses on the origins and resources of health-promoting and interprets states of well-being as a continuum ranging from absolute health to profound disease states. The central concept of the salutogenic model is the sense of coherence (SOC) and it is conceived as a global orientation that expresses the degree to which one has a pervasive, enduring yet dynamic sense of confidence that: (1) the stimuli coming from the internal and external environment in the course of life are structured, predictable and explainable (comprehensibility); (2) the resources are available to one to meet the demands posed by these stimuli (manageability); and (3) these demands are challenging, worthy of investment and commitment (meaningfulness) [34]. SOC is a key concept in the development of resilience under adverse circumstances [35,36,37].

In turn, the salutogenic model is composed of a second construct: *generalised resistance resources* (GRRs). GRRs are defined as the set of personal, social or contextual resources and factors that minimise or counteract the stressors of everyday life by facilitating adaptation and coping in the face of adverse life circumstances [38]. Although not conclusive, research indicates that there is mutual feedback between SOC and GRRs, favouring well-being and health, which means that the use of GRRs contributes to a strengthened SOC and, likewise, a strengthened SOC implies a better use of GRRs [39,40].

However, it is well established that stressors negatively affect the state of health and well-being. In this regard, in [34,41] stressors are grouped into three broad groups: chronic stressors, major life events and daily hassles. This research focuses on the second group of stressors, understood as life events of negative impacts, which provoke a homeostatic disequilibrium on people’s well-being at some point in their life cycle [31,32,33,34,35,36,37,38,39,40,41,42].

The characteristics of the university environment and the confluence of emerging adulthood [43] make it possible for young university students to be subjected to various stressors, such as the exploration of professional identity, family independence, a greater degree of autonomy and responsibility, exacting academic demands, or family and personal expectations [44]. Furthermore, a study by the World Health Organisation on the prevalence and distribution of mental disorders in first-year university students revealed that 1 in 3 students (31.4%) suffered some mental disorder in the first academic year at university and in 1 out of 5 cases (20.4%) the disorders caused developmental problems, not only at the academic level but also affecting social life, personal relationships and work [45,46]. In the Spanish context, knowledge about how stressors affect the well-being of university students is not a widespread field of study, although some studies do exist. In relation to the prevalence of major depression, in [47] depression is reported to be highly prevalent in a sample of university students, with 8.7% of major depressive episodes, with the most common symptoms being a depressed mood (81.3%) and sleep disturbance (79.2%). In a more recent study of female university students, the results showed that 18.1%, 22.8% and 13.5% had severe/very severe levels of depression, anxiety and stress, respectively [48]. Along the same lines, it is concluded in [49] that one third of Spanish university students report having suffered from a common mental disorder in the last year, and one third of them report a severe impairment of their functioning. In addition, it is found in [50] that 21% of students persist in suicidal ideation one year after starting their university studies.

This research indicates that the university stage is a critical period for the transition to adulthood that is not free of stresses, which undermine students’ health and well-being, both the well-being of students and their entrepreneurial performance in the development of their life projects. While there is research on resilience and coping with stressors in the university context [51,52,53,54], there are no studies on entrepreneurial resilience, in the sense of personal growth and development, but rather directly related to the organisation and creation of companies [55]. Personal growth as an entrepreneurial process is linked to the well-known concept of “entrepreneurial orientation” [56,57,58,59], which in turn is associated with performance theory [60]. This theory includes cumulative research on strategy and refers to three dimensions: innovation, risk-taking and proactivity [61]. Applied to individuals (it could also be applied to organisations), the process of entrepreneurial personal growth adds to the mere resilience of those strategic components that make up a specific disposition towards entrepreneurial behaviour, related to habitual tasks and the necessary adjustments required by the contexts in which the action takes place [62]. In a way, personal entrepreneurial growth highlights, as with the entrepreneurial orientation construct [63], the links between entrepreneurial attitudes and entrepreneurial behaviours [64,65]. In the academic environment, in which we have conducted the research, we could add some other strategic dimensions that characterise the process of personal entrepreneurial growth: self-confidence (confidence in relation to certain personal attributes), autonomy (ability to act with one’s own criteria and independently), perseverance (perseverance in achieving goals), achievement orientation (preference for taking responsibility and the submitting to evaluation of results) and problem solving (a process for dealing with difficulties). From a salutogenic perspective, the aim of this research is to explore the strategic components of the entrepreneurial profile of ten young university students and how they have shaped their entrepreneurial resilience after experiencing negative life events (NLEs).

## 2. Materials and Methods

### 2.1. Research Design 

This research is a multi-case study [66,67] that was developed through a mixed methodology [68] using quantitative (RS-14 test) and qualitative (interviews) instruments. Bibliometric research [69,70] reveals that mixed research methods are widely used in entrepreneurship studies and corroborates their methodological effectiveness, irrespective of the research sequences implemented [71,72], as they enable: (1) a more comprehensive view of processes and results, (2) an increase in validity, (3) an improvement in the interpretation of results and (4) the generation and integration of more detailed and explanatory knowledge [73,74,75]. The research design was based on the principles of priority and sequentiality, with the qualitative method being primary (QL) and the quantitative method secondary (qt). The research frequency was quantitative and qualitative. Thus, the study was structured in two research phases: qt→QL [76]. The quantitative phase (1) was implemented through a descriptive and selective non-experimental study [77] to determine the degree of resilience of the participants and to select the subjects with the highest levels of resilience. The qualitative phase (2) and central core of the research was developed under the phenomenological and interpretative approach [78], based on the exploration, interpretation and narrative understanding of the participants’ life experiences, through the memories and reasoning of their own personal trajectories [79]. In our case, this approach aims to interpret and understand how people give meaning and significance to NLEs in the process of building entrepreneurial resilience. Interventional studies involving animals or humans, and other studies that require ethical approval, must list the authority that provided approval and the corresponding ethical approval code.

### 2.2. Sampling and Participants

In order to select the participants, a non-probabilistic convenience and purposive sampling was used [80], following these inclusion criteria: (1) the participants belonged to the Pedagogy and Teaching Degrees of the University of Seville, (2) they had identified at least one NLE according to the Life Events Checklist for the DSM-5 [81] and (3) they had obtained very high scores (90–99) on the Resilience Scale (RS-14), the resilience measurement instrument. Following the sampling cycle [82], the participant sample (quantitative phase) consisted of 276 subjects who completed the RS-14 test and the real sample (qualitative phase) consisted of the 10 participants with the highest scores on the RS-14 test.

### 2.3. Data Collection Tools

The data collection instruments selected in this research are disaggregated according to the phases of mixed research (qt-QL).

Quantitative phase (qt): the level of resilience was measured with the Resilience Scale (RS-14) [83], validated in Spanish [84]. This consisted of 14 items with a 7-point Likert scale (“completely disagree” = 1, “completely agree” = 7). The SR-14 was made up of a single factor (resilience), reflecting the original dimensions of *Personal Competence* (11 items) and *Acceptance of self and life* (3 items). The psychometric properties of the RS-14 are adequate with an overall scale internal consistency (α 0.79) [84].

This scale was used for the selection of participants under the following criteria: (1) the scale was validated by Spanish university students showing adequate internal consistency (α 0.79) and robust validity with significant correlations with the resilience scale CD- RISC [3]. (2) The RS-14 was used in different studies in university populations [85,86]. (3) Previous studies were carried out with this resilience scale in the entrepreneurial field [87,88]. (4) In the Spanish context, there is a lack of instruments that measure the intrapersonal factors of entrepreneurial resilience in the university population. In this respect, in the initial instructions for completing the scale, the following indication was introduced for the completion of the items: “As an entrepreneurial person I consider that…”. In this way, participants responded to the SR-14 from an entrepreneurial perspective, as in other studies on resilience and entrepreneurship [89]. These criteria ensure the validity of the choice of SR-14 for this study.

Qualitative phase (QL): Understanding how entrepreneurial resilience is formed was studied through in-depth interviews based on a semi-structured script [90]. The development of the first interview script was based on the available literature [91,92,93]. Subsequently, the interview script was subjected to the judgements of ten experts who analysed its validity and relevance. The experts were selected for their recognised academic and professional backgrounds in the field of resilience and were drawn from the academic and therapeutic sectors. In the academic sector, 6 experts with at least 2 publications on resilience in indexed journals were selected, and in the therapeutic sector 4, professionals with at least 5 years of experience in the development of therapies were chosen. Finally, the interview script was comprised of three thematic blocks with a total of 87 questions: personal events (11 questions), resilience (41 questions), and resilient entrepreneurial identity (35 questions). An interview is the instrument used to characterise the entrepreneurial profile of the resilient people selected in the quantitative phase. An interview allowed us to discover the strategic components that predispose towards entrepreneurial behaviour and how they were formed.

Overall, the application of the SR-14 in the quantitative phase only involves the selection of highly resilient participants. In the qualitative phase, we used the interview as the main instrument of information collection to map the strategic dimensions that characterise personal entrepreneurial growth and the processes of how entrepreneurial resilience was achieved. In this way, the qualitative data collected will be crucial to define the strategic dimensions of the entrepreneurial personal growth process.

### 2.4. Data Collection 

Prior to the beginning of the research, authorisation was requested from the Dean of the Faculty of Education Sciences to carry out the study. Subsequently, an e-mail was sent to the teaching staff of the Bachelor’s Degrees in Pedagogy and Primary Education explaining the aim of the research and requesting access during their teaching hours to collect data using the RS-14 scale (quantitative phase). A set of twelve teachers agreed to collect quantitative data from both degrees. The data collection took place during the month of October 2020 and the students were informed of the purpose of the research, of their voluntary participation and of the confidential and anonymous processing of data for exclusively scientific use. The participants in the quantitative phase gave their written informed consent. Once the data from the RS-14 scale had been collected, the data were emptied and the subjects were classified according to the level of resilience of the total scores obtained on the RS-14 scale into: very high (90–99), high (80–89), normal (70–79), low (60–69) and very low (50–59). In the qualitative phase, the 25 participants with the highest scores on the RS-14 scale were selected. They were then contacted by e-mail for in-depth semi-structured interviews. Of these 25 potential participants, 19 replied affirmatively to participate in the qualitative phase. However, nine of them finally declined to participate in the interviews for work and personal-related reasons. The interviews were carried out by the first author during the month of November 2020 in an office of the Faculty of Education Sciences. Prior to the interviews, the participants were informed about the purpose of the interviews and the scientific procedure to be followed in the data processing. The interviews were recorded and lasted between 60 and 90 min each, ending when there was a saturation of data [94].

### 2.5. Data Analysis 

In the quantitative phase, a descriptive percentage analysis and a study of the mean scores obtained on the RS-14 scale were carried out using the SPSS version 27 (IBM Corp, Armonk, NY, USA) programme. From phenomenological and interpretative epistemology, the qualitative data were analysed via thematic analysis. This method of analysis “is not wed to any pre-existing theoretical framework and so it can be used within different theoretical frameworks (…). Thematic analysis can be an essentialist or realist method, which accounts for the experiences, meanings and realities of the participants” [95]. The thematic analysis of the data provided by the interviews followed the phases described in the methodological guide of [95]: first, the interviews were transcribed verbatim by two researchers, and each interview was listened to at least three times, achieving a high degree of accuracy in the transcriptions. Subsequently, a third researcher checked all the transcribed interviews for accuracy. Second, a process of reading and re-reading the interviews was carried out and two independent researchers generated the initial codes in relation to the semi-structured interview script. Once the first three interviews had been coded, together with a third researcher, the list of codes was agreed upon by eliminating, reformulating and adding certain codes. The coding was carried out with the Atlas.ti. 7.0 programme (Scientific Software Development GmbH, Berlin, Germany). Third, once the list of emerging codes was compiled, potential themes related to the “lived experiences” of entrepreneurial resilience were identified and organised into a structure of main themes and sub-themes based on their prevalence and representativeness. Fourth, the researchers reviewed and agreed on the selection and organisation of the themes and sub-themes.

Finally, the thematic structure was defined and nominated, i.e., the textual excerpts of each theme were identified and the essential aspects that would integrate each theme and sub-theme were determined. To do this, the themes themselves and their relationship with the other themes were considered. Through this process, the NLEs were interpreted and understood. The quality of the research was ensured by applying the following procedures [96,97]: (1) credibility was ensured by in-depth revision of the data, along with comparing data between researchers (triangulation); (2) dependability was ensured by using participant-identifying keys and describing data analysis techniques; (3) transferability was maintained by using purposive sampling, as well as describing the participants and data collection in detail; and (4) confirmability was obtained by analysing the data with Atlas.ti. 7.0 and checking the information from the interviews with the participants.

### 2.6. Ethical Considerations

This research was approved and reviewed, including ethical aspects, by the committee of the Spanish Ministry of Science and Innovation and the State Research Agency of the Spanish Government under reference number PID2019-104408GB-I00.

## 3. Results

### 3.1. Quantitative Study

#### 3.1.1. Socio-Demographic Characteristics of the Participants

Two hundred and seventy-six students from the Faculty of Education Sciences at the University of Seville participated in the study by completing the RS-14 scale. The socio-demographic characteristics of the participants are shown in Table 1. The participants have a mean age of 21 years (SD = 3.20) and the age range is between 18 and 45 years. The highest percentage of the participants are female (87.7%), compared to a lower percentage of males (12.3%). This very disparate percentage distribution between men and women is a consequence of the feminisation of the degrees in Education Sciences in the Spanish university context [98]. Data from the National Institute of Statistics show that in the academic year 2020/21 the percentage of women enrolled was 77.8% compared to 22.2% of men. Similarly, this distribution also occurs in the Faculty of Education Sciences at the University of Seville, where the total number of women enrolled corresponds to 71.68% as opposed to 28.32% of men. The composition of the sample by year was as follows: first year (54.7%), second year (14.1%) and fourth year (31.2%). As for the distribution by degree, the percentages obtained were 41.3% in primary education and 58.7% in pedagogy.

#### 3.1.2. Participants’ Responses Related to Resilience Level

The RS-14 scale consisting of fourteen items was used to assess the students’ level of resilience, as can be seen in Table 2. High scores were obtained for all the items studied, the mean being between 4.03 and 6.29. Among the high scores, items 14 (“in an emergency, I am someone people can trust”) (42.4%), 13 (“I am proud of the things I have achieved”) (44.9%), 12 (“I can usually find something to laugh about”) (47.8%) and 11 (“my life has meaning”) (48.9%) stand out. In contrast, an increase in low-scoring responses is observed for items 1 (“I generally take things in my stride”) (15.9% and 19.9%), 2 (“I am not afraid of difficulties because I have experienced them in the past”) (8.7% and 12.7%) and 3 (“I am a person with adequate self-esteem”) (7.6% and 12.2%). The rest of the items have high mean scores, approximately between 4.5 and 5.5.

#### 3.1.3. Resilience Levels

The resilience scores were distributed into the following categories: “Very low (50–59 points)”, “Low (60–69 points)”, “Normal (70–79 points)”, “High (80–89 points)” and “Very high (90–99 points)”, as shown in Table 3. The majority of the participants, 64.5%, obtained an overall score on the scale within the value “High”, showing a positive perception of their level of resilience. Furthermore, 26.4% of students scored in the “Very High” range, while only 9.1% was in the “Normal” range. Therefore, the majority of the students agreed that, in general, they had a high level of resilience.

### 3.2. Qualitative Study

#### 3.2.1. Demographic Characteristics of the Participants

Qualitative data were obtained through in-depth interviews and were analysed comprehensively, adopting a biographical narrative approach. Ten participants were interviewed, as summarised in Table 4. The participants scored between 90 and 98 points on the RS-14 scale. The majority of the participants were women (7), compared to a small number of men (3). The mean age of the participants was 26 years (SD *=* 4.9), the age range being between 21 and 37 years. With regard to undergraduate degrees, the majority were in pedagogy (7), compared to those in primary education (3). The participants were enrolled in the fourth year (5), second year (1) and first year (4). Finally, among the Negative Life Episodes (NLE) they experienced, family problems and illnesses stand out.

#### 3.2.2. Shaping Entrepreneurial Resilience

The information from the interviews indicates the set of processes implemented after the emergence of the NLEs. The structural thematic analysis was composed of two main themes and four sub-themes, respectively, categorised into intrapersonal and exopersonal (Table 5):

#### 3.2.3. Resilience Shaping Processes

The resilience of young university students was shaped by intrapersonal processes, such as cognitive, emotional and behavioural processes on the one hand, and exopersonal processes, such as social support, on the other hand.

##### Cognitive Processes

The *perception* of the stimuli as NLEs was generalised across all the participants. The assessment of the severity of adverse events is unique to personal subjectivity and is closely related to the availability of the person’s resources. When the demands of hazards exceed personal resources, adverse experiences are evaluated with a high degree of severity. The university students interviewed describe that in the face of adverse experiences, such as sexual abuse, terminal illness, abandonment or family breakdown or the death of parents in accidents, they have not had sufficient resources to manage NLEs. Participant S10P90E21SM narrates that she has *“suffered sexual abuse by a very close relative, from the age of 6 to 14 (…) I didn’t know what to do, I didn’t know what was happening and I didn’t know how to act, I was a child who didn’t understand anything”*. S6P92E23SV describes the lack of resources to adequately manage her NLE: *“I witnessed my father beating my mother and verbally humiliating her on many occasions, ad nauseam. I also experienced my brother’s schizophrenic crises, which were brutal. And later, I had to take care of my mother who suffered from bipolar disorder. All this pushed me beyond my limits, I couldn’t cope any more”.* Similarly, the rest of the participants described the lack of personal resources and their perception of the severity of their adverse experiences as NLEs.

According to the narratives, the NLEs that occurred are unchangeable and uncontrollable situations, i.e., there are no opportunities to change them. Faced with this, the participants opted for *cognitive accommodation*, since it was impossible to change the adverse situation that threatened their aspirations and life goals. S2P94E25SM indicated that *“I have resigned myself, there is no way out, I know that this disease is going to end my life, at most in a few years (…) I have had to adapt”*. The limited mental resources of the participants allowed them to exercise a high degree of cognitive control. In this sense, they focused on relevant information with the intention of reducing the uncertainty of the severe adverse situations they were experiencing. S8P91E22SM’s cognitive control reveals how he implemented cognitive control to minimise harm to his mental well-being.:

*“I was saturated, everything in my mind was totally confused, too many things had happened to me that I wasn’t able to assimilate, I was paralysed, I couldn’t make a decision to move on with my life. There came a time when I had to act and I started to focus on what was important. Everything I did and thought about was directed towards what really mattered to me.”.*

Cognitive flexibility has been a constant in the participants’ response to the changes that occurred in their context due to the emergence of the NLE. This is expressed by S4P92E26SV, “*… my way of thinking changed, I had to look for new solutions to the problem*”, and S3P93E32SV, “*… the situation was serious, everything I thought did never work, I had to reflect and see new ways to get out of that situation*”.

##### Emotional Processes

The intensity of the emotions that arise in NLEs has led the participants to describe profoundly negative emotional states. In some cases, these emotional states have involved acute stress reactions leading to emotional blockage. Thus, S7P91E25SM recounts that *“when I was told that the tumour was inoperable and the time I had left, I was in shock because I was not able to do anything. I was in shock because I was not able to assimilate it”*. However, sometimes traumatic experiences leave permanent after-effects. The emotional intensity that emerged in the NLEs decreased as time passed, and the participants adapted to their new life circumstances. In this sense, some participants stated that they did not feel emotional blocks, although they did experience episodes of anxiety and depression after the NLEs: *“I had that depression, when everything came to light”* (S5P92E24SM) or *“I also had some anxiety attacks”* (S9P90E30SM).

Undoubtedly, NLEs have involved the emergence of high intensity negative emotions that have affected, in one way or another, the emotional balance of the participants. However, despite the impact suffered, the functioning of the emotional system has not suffered a serious disruption that has caused irreversible damage to the well-being of the participants. On the contrary, emotional intelligence has been a mediating variable between the NLEs and their consequences on the health and well-being of these young university students.

Thus, despite the initial emotional confusion, all the participants indicate that they were gradually able to identify how they felt and were able to define their emotional states: *“I was very clear about how I felt”* (S1P98E37SM) and *“I knew how to detect anger, strength, hope, rage, even revenge”* (S9P90E30SM). Although, the lack of personal and emotional maturity in childhood and pre-adolescence has made it difficult to identify and define certain emotions or feelings when the damage has occurred in these stages: *“when I started therapy, because of all the abuse, the psychologist helped me to put my mind in order and name the emotions I felt. From then on, I got to know and identify my feelings and emotions”* (S7P91E25SM). The emotional states they experienced ranged from negative emotions, such as sadness, anger, pain and frustration, to positive emotions, such as hope, optimism or joy:

*“Sadness, impotence. Sometimes anger. Pain”*(S1P98E37SM).

*“The sadness was very obvious, and so was the loneliness. I felt frustrated, that I couldn’t do any more, I felt powerless, I was falling down, I was tired of crying”* (S3P93E32SV).

*“Hope that everything would pass”* (S6P92E23SV).

Not only emotional awareness, but also emotion regulation is key in the whole process of repairing emotional states after NLEs. Distorted thoughts and protracted episodes of rumination or feelings of guilt had an impact on the decrease in well-being. In the face of these anxiogenic states, the participants managed their emotions, although this was not an efficient and continuous process, but was rather characterised by fluctuations in emotional management. On the whole, however, they were able to regulate the emotional load positively affecting well-being, as S10P90E21SM says: *“It depends on the day. There were days when despite all the bad things I was able to lift my spirits, to be positive and strong. However, there were also days when I would sit down and cry and I couldn’t stop the pain”*.

The expression of moods was also part of the emotional processes. Nine of the ten interviewees openly indicated that expressing their emotional states was key to counteracting the negative emotional impact of NLEs. In one way or another, the participants described emotional expression as a means of venting, comfort and consolation:

*“I cried a lot. I used to vent to my friends (…) Talking about it helped me to let off steam.”* (S2P94E25SM).

*“Talking about them. Accepting them”* (S8P91E22SM).

*“I write about what I feel, it helps me a lot”* (S4P92E26SV).

##### Behavioural Processes

The participants responded to stressful or anxiogenic situations through a range of coping strategies. Thus, the coping implemented was not characterised by avoidance. On the contrary, the behaviour of all the participants was aimed at reducing the stressor as much as possible, investing a great deal of personal effort to achieve this. The participants neither denied the problem nor avoided it, which allowed them to generate behaviours aimed at solving the problem. S5P92E24SM tells us that:

*“And that’s what I grabbed on to when I got caught in that loop (…) I realised that the damage was already done, and that I had no choice but to rebuild myself. I changed my attitude and everything started to get better”.*

In general, the participants indicated that, at first, the thoughts that generated the NLEs were characterised by their distortion and dysfunctionality, making negative cognitions possible. In this situation, the participants re-evaluated and reinterpreted the adverse situation, i.e., they sought the positive and optimistic meaning of the stressor. S2P94E25SM expressed the following:

*“When it all started, my thoughts were self-destructive, nothing that was happening made sense, I just wondered: why me? As time went by, my point of view changed, I tried to look for the good side”.*

This reinterpretation implies a recognition and acceptance of the adverse events under a positive approach to the situation. As participant S4P92E26SV says: *“… although I couldn’t believe it, I accepted it, I think that was the hardest thing to do, to accept that I had a serious problem that was affecting me deeply and could have serious consequences for my future”*.

##### Social Support Processes

All the participants consider that they received help from one or more people around them. Moreover, they think that social support is essential to cope with adverse situations. Social support becomes a reference that facilitates a balanced transition from the adverse situation. S6P92E23SV expresses the social support of his environment:

*“… my wife and her family (…) I saw the kind of life they led and then I said to myself that I was lucky to see that reference and I wanted to follow that model (…). I had a special friend, we have a lot of affinity. He lived with a good family (…). They took me in. That help is very important in my life and it also left a big mark on me. It helped me a lot, in terms of food, money, clothes, etc.…”.*

Three of the participants explicitly sought this social support from mental health professionals, as they perceived the need for professional counselling to address NLEs. As S10P90E21SM recalls: *“The key help was my psychologist, she gained my trust and the therapy was great”*. The other seven participants indicated the presence of social support, although they did not intentionally seek it, but rather found it in their environment. Thus, social support is not linked to mental health professionals, but to people close to them who offered support, care, affection and security. These people established strong emotional bonds with the participants:

*“…the schoolteacher saw great potential in me and, in a way, took responsibility for me when I was 11 years old, she was the one who helped me to get up every day with the strength of will to attend classes and not to sink. She was always there, offering me her support and affection”* (S9P90E30SM).

#### 3.2.4. Strategic Entrepreneurial Dimensions

In the processes of shaping resilience, the participants acquired a set strategic entrepreneurial dimensions promoting personal growth. The students interviewed believe that they acquired a good degree of self-confidence and claim to have the ability to overcome adversity successfully. As stated by S7P91E25SM: *“I think I am confident enough to overcome any situation, but I also think that you never know what you might experience”*. In turn, the participants presented a high degree of autonomy, as they were able to satisfy their basic needs, act with judgement and acquire personal independence following their own values: *“Yes, I am used to being independent and autonomous (…) because I have had to do things by myself and make very difficult decisions to move forward in life”* (S1P98E37SM).

According to the participants, the new circumstances following the NLEs required their initiative and proactivity, that is, they had to perform certain behaviours of their own accord, anticipating certain problems and their possible consequences. The participants’ behaviour was not driven by external factors; they self-directed their behaviour towards the goals they set for themselves. The initiative of S8P91E22SM was clearly revealed when she stated that *“I don’t want to let things just happen but try to promote them myself. I like not to be satisfied and to find more ideas and solutions to problems”*. Yet, six participants confirmed the personal efforts made to gain this degree of autonomy: *“It has not been easy, everything has involved a lot of effort and work on my part, nobody has given me anything for free”* (S5P92E24SM).

All the participants agree that coping has made them more perseverant. Four of the participants confirmed that before the NLEs, they did not possess such levels of perseverance to achieve the goals they set for themselves. The rest of the participants stated that their perseverance became stronger and stronger after the adverse experiences. S3P93E32SV and S10P90E21SM, respectively, expressed the degree of perseverance they possess: *“Before I was not like that, after my bad personal experiences I have become more persistent and constant”* and *“I think I have always been constant. It is one of the things that helped me to overcome everything I went through, but now I am even more so”*.

The participants agree that they offered original proposals to solve the various difficulties they encountered during and after the NLEs. In this sense, the narratives indicate that the creative processes made it easier for the participants to better adapt to different contextual demands. Similarly, they describe in their narratives that providing creative solutions enabled them to change adverse contextual situations for their well-being. Two of the participants describe it in the following way:

*“I have developed creativity to solve any kind of problem”* (S2P94E25SM).

“*You have to be creative (…) to react well to such a difficult situation*” (S6P92E23SV).

It is common for the participants to consider that the impact and management of NLEs has meant for them, above all, acquiring the capacity to solve problems of different kinds, whether they come from the adverse situation itself or as a consequence of it. As S5P92E24SM tells us:

“*When you have a problem and you make a decision and you carry it out, you solve the problem, but when you don’t solve it and the days go by, other types of problems come up that need other solutions. I always say the same thing, a problem appears, I find a solution*”.

In general, the participants corroborate that they learned to solve their problems regardless of their size, looking for the most efficient solutions, although again the personal efforts needed to solve the problems are evident in the narratives of five participants:

“*After what I have been through, I can solve any problem, improvise solutions or get out of any problem by myself, although I also know that it takes a lot of will and effort to remove the problem*” (S4P92E26SV).

#### 3.2.5. The Process of Future Projection of the Self

##### Personal Goals and Efforts

Two participants with serious illnesses primarily set themselves health-related goals. On the one hand, S3P93E32SV reports that “*The most important thing is to be in good health within the limits that this disease allows me. I try to do everything the doctors say to continue with my life as best I can*”. On the other hand, S8P91E22SM indicates that “*it is essential to endure the treatments and then move on when my body recovers from the side effects, although I’m always in that circle of stopping and then starting again*”. Regardless of the illnesses, all the participants have goals related to the completion of their university studies and their incorporation into the labour market in the field of education: “*I have focused on studying hard to get good grades and to be a good educator*” (S1P98E37SM), and “*I mustn’t fail subjects, I want to get to the final year and have my teaching degree, I want to work*” (S9P90E30SM).

After experiencing NLEs, the participants report that an overriding issue in their lives is to achieve a good degree of physical and mental well-being. The narratives indicate that their understanding of life is closely linked to this goal of well-being: “*After all the pain I have gone through, I always prioritise having inner balance, feeling good inside, leaving the old ghosts behind me*” (S4P92E26SV). At the same time, they coincide in their interest in generating and maintaining quality relationships with the people around them: “*I love making friends and having friends, friends bring you a lot, they help you and you can help them*” (S7P91E25SM).

The students reveal that achieving their goals is not without effort and personal commitment. According to them, perseverance towards their goals comes, on the one hand, from knowing what their priorities in life are in the short, medium and long term and, on the other hand, from the skills they acquired after overcoming the NLEs: “*I have learned to know what I want and to set goals to move forward little by little. Now I am stronger, I have more inner energy*” (S1P98E37SM). Although they make strong efforts, the participants expressed that this determination has a personal origin. Subject S8P91E22SM clarifies that: “*the energy comes from within me, it’s okay to support from outside, but the engine is inside of you*”. Similarly, the students feel confident and empowered to achieve what they set out to do: “*I rely on myself and all the things I have learned to get where I want to go*” (S4P92E26SV). The students agree that efforts are fundamental in the achievement of personal goals. However, they specify that it is also necessary to count on the help of people close to them and to have certain material resources, as S5P92E24SM says: “*primarily you, but there are also your friends or family members and, of course, having some minimums to live on*”.

##### Personal Projects

The NLEs entailed profound changes in the lives of these university students, since they had to trace life trajectories based on the new circumstances. In some cases, the occurrence of NLEs implied an abrupt rupture of their previous life projects. In other cases, personal projects had to be drastically reorganised due to the life transformations that the participants underwent. In one way or another, the participants had to take risks in the uncertainty of building a new life. As stated by S10P90E21SM: *“Life totally changed what I wanted to do with my future";* and S6P92E23SV: *“when everything happened, I realised that I had to change a lot if I wanted to move on with my life".*

All the participants corroborate that the construction and development of personal projects after the NLEs made them aware of how to build personal projects. Thus, S7P91E25SM states that *“before, I was not aware of how my life was going towards the future, towards things just like that. Now, I stop to think about what I want, how I am going to do it, how much time I will spend on it or what things I will need”*. The students describe that, once they overcome NLEs, their lives focus on certain priorities, such as developing and maintaining physical and mental well-being, attaining an adequate education and entering the labour market, or fostering fruitful personal relationships with the people closest to them:

“*I like to feel good about myself, it’s something I don’t mind spending my time on*” (S2P94E25SM).

“*I want to finish my university studies and become a teacher. It is my greatest wish*” (S9P90E30SM).

“*I plan to continue my studies, but also to spend as much time as possible with my loved ones*” (S5P92E24SM).

In general, the narratives reveal that the projects emerge from personal values and have a high relevance for the participants’ lives. At the same time, they agree that they enjoy the implementation of the projects and are deeply involved in them: *“I think that the important thing is to be happy and to be well with others. Everyone should know what they want to do with their lives and do it in accordance with their personal values (…) so it makes more sense and you get involved in it”* (S3P93E32SV). Each of the participants is developing a variety of personal projects, in different degrees, but they corroborate that they are capable of implementing and executing them adequately, although they are aware of how contextual variables can influence their execution: *“I see myself capable of doing what I have set out to do in the medium and long term, but of course I can’t control everything that happens, although I try to make sure that everything goes in the right direction”* (S5P92E24SM).

To the same degree, all the participants consider that the construction of their personal projects is having a positive effect on their lives, making them feel more fulfilled and hopeful: *“I feel very happy, I see the future with enthusiasm and positivity”* (S9P90E30SM). Similarly, the personal projects they described have a purpose related to entrepreneurial personal growth, proposing projects of various kinds, but always with a genuine personality development aspect. Thus, the subjects set themselves second-order personal projects linked to their personal values and life purpose, together with first-order personal projects related to the medium and short-term execution of intermediate goals: *“Like everyone else, I want to be happy (…) It’s like a path where you move forward according to the goals you set yourself, but without losing sight of the fact that I want to be good in everything I do*” (S4P92E26SV). 

Finally, the participants indicate that the strategic entrepreneurial decisions learned during the NLEs they experienced are valuable personal resources that facilitate the construction of their personal projects: *“Everything I have learned on a personal level is helping me to be able to generate all my desires”* (S3P93E32SV).

## 4. Discussion

From a salutogenic perspective, the aim of this research was to explore how ten young university students shaped their resilience and entrepreneurial capacity after they experienced negative life events (NLEs). This study was implemented through mixed research, where the quantitative phase was used to select the cases with the highest degree of resilience and the qualitative phase to know the strategic entrepreneurial components involved in resilience, and to understand how the entrepreneurial resilience of the selected university students is formed under a phenomenological and interpretative approach. In the quantitative phase (qt), the data indicate that the university students in the sample possess acceptable levels of resilience. The surveyed participants range in resilience from normal (9.1%) to very high (26.4%), with 64.5% being in the high levels. No participants are in the very low or low levels. Other studies on Spanish university students show similar results with levels close to 60% in high resilience and 14% with strong resilience, although, on the contrary, they do show low levels of resilience at 30% [99]. Studies in Italy [100], Germany [101] and India [102] show fluctuating levels of resilience in emerging adulthood during the university stage. This explains the emergence of qualitative and mixed research trying to understand how resilience develops and consolidates itself in adulthood at university [103,104].

In the qualitative phase (QL), the data indicate that entrepreneurial resilience is configured through three moments: (a) the impact of the NLEs, (b) learning to overcome problematic situations and (c) the future projection of the self. In these three moments, university students used both SOC components and intrapersonal and exopersonal GRRs as promoters of entrepreneurial resilience.

Moment one is the impact of the NLEs and is characterised by the occurrence of the adverse situations and the subject’s management to minimise the stressors and their possible damage. At the moment of impact, the participants managed to implement the joint functioning of cognitive, emotional, behavioural and social support processes. The processes of cognitive perception and emotional identification allowed the interviewed university students to achieve a good degree of comprehension of the severity of the NLEs and the emotional states derived from these adverse experiences. On the other hand, accommodation, control and cognitive flexibility, together with the regulation and expression of emotional states fostered the participants being able to obtain an adequate manageability of the anxiogenic situations generated. In addition, cognitive emotional functioning supported coping behaviour in the face of NLEs through a focus on problems, emotions and meaning [105]. This cognitive, emotional and behavioural triad would be intrapersonal GRRs that promote entrepreneurial resilience. Previous studies [40,106] corroborate that cognitive, emotional and behavioural mechanisms are intrapersonal resources that strengthen SOC. However, we also find contextual GRRs, such as the social support received in the narratives. In this regard, the participants identify the figure of the resilient mentor [107], both from an explicit perspective of seeking social support, especially in mental health professionals, and from an implicit perspective, where the participants casually found social support in the people close to them [108].

NLEs are anxiogenic situations that are highly likely to affect well-being. Nevertheless, studies indicate that sometimes these experiences become personal situations of coping and sustaining future well-being [109,110]. In line with the other researches [111,112], we posit that intrapersonal and exopersonal GRRs recognised by the participants would strengthen SOC through learning acquired in NLEs [41]. This is the second moment that the participants identified.

The overcoming moment refers to all the strategic entrepreneurial dimensions acquired by the participants once they faced the NLEs. Thus, the moment of overcoming the NLEs would be characterised as a situation of reflexive and experiential learning, where the participants actively thought about the causes, consequences, possibilities and personal contributions that the experiences of the NLEs have brought to them [113]. Likewise, this second moment could also be characterised as an educational overcoming, since the participants, through their memories and autobiographical reasoning, were able to better understand the adversities they experienced and acquire a constructivist vision of the NLEs.

In this way, university students were able to identify a range of strategic entrepreneurial dimensions. The qualitative data indicate that resilient processes incorporate certain strategic dimensions of entrepreneurial behaviour with a different degree of concreteness. Thus, at a first level, we obtain two basic dimensions of entrepreneurial orientation: innovation or creativity and proactivity or initiative. At a second level, there are specific strategic dimensions, such as autonomy, perseverance, problem solving and self-confidence. This set of strategic entrepreneurial dimensions would be appropriately constituted as intra-personal GRRs for the personal future after the NLEs. [114]. Although they all have their degree of importance, self-confidence as a central element in the salutogenic model takes on a preponderant relevance, since the components of SOC, *comprehensibility, manageability* and *meaningfulness*, are mediated by the degree of confidence in recognising stressors, coping with the demands of those stressors through the available resources and the commitment to overcoming challenges [115].

In the third moment, the future projection of the self underlies the question of what happens when NLEs are successfully faced and overcome. In general, a wide range of new possibilities opens up for the participants. The experiences of the university students interviewed indicate that there is a profound transformation of the system of beliefs, values, attitudes and behaviours prior to the NLEs. According to them, this internal change allowed them to observe new opportunities and achieve a life with a greater degree of *meaningfulness*. Previous research explored how negative experiences promote a meaningful life [116,117].

The third moment is characterised by the implementation of the strategic dimensions of entrepreneurial behaviour, enabling the projection of the future self [118] and the construction of life projects [119], through personal goals and efforts [120]. However, after the moment of educational impact and self-improvement, the participants explicitly manifested the acquired entrepreneurial strategies, it is no less true that, in the third moment of future projection of the self, they developed strategic entrepreneurial dimensions related to risk-taking (basic level) and achievement orientation (specific level) for the achievement of their objectives, goals and personal projects. It would be difficult to understand the implementation of personal projects without a certain degree of risk-taking and achievement orientation. With the development and implementation of the strategic dimensions, the participants built up a personal goal structure, differentiating between focal and instrumental goals [121]. Both types of goals were constituted by the participants through intrinsic and identified motivations, i.e., they chose the goals and objectives that represent their system of beliefs, values and attitudes [122]. These are so-called self-concordant goals [123]. Recent research corroborates how the choice of personal goal concordance generates optimism and well-being [124]. The self-concordant goals selected by the participants are linked to: first, maintaining and developing personal well-being; second, academic or professional development; and third, fostering quality inter-personal relationships. The achievement of these objectives implies a deep commitment on the part of the participants, which is reflected in the personal efforts they made towards their attainment. Underlying these personal efforts are the strategic dimensions acquired during the moment of self-improvement. In spite of their personal efforts, the participants are aware that there are contextual resources, which are convenient in order to achieve their goals, and material and financial resources. 

Distinguishing between focal and instrumental goals [121] also implies the difference between first- and second-order projects [125]. In the participants’ narratives, we can distinguish how they elaborated first-order projects to achieve certain instrumental goals that would facilitate the development of second-order personal projects, which are aimed at focal goals or self-concordant objectives. Thus, the participants construct their personal projects hierarchically. Studies on Spanish university students support this hierarchical conception of personal projects in emerging adulthood [126]. In this sense, it is common for all the participants to establish a hierarchy, where second-order projects, which guide first-order projects, are defined as being directed towards personal development, growth or well-being. It is not surprising that the effect of the personal projects constituted after the NLEs on well-being is considered as positive by the participants. This notion of the positive effect of personal projects was indicated by early studies on life satisfaction and the organisation of personal projects [127]. Finally, in the projection of the self through personal projects, the participants recognise the value of the strategic dimensions of entrepreneurial behaviour acquired in the implementation of their personal projects. In this sense, all the narrative data indicate that the implementation of intrapersonal GRRs, in this case, the strategic dimensions, enable a more solid configuration of the SOC. The described strategic dimensions favour a better *comprehensibility, manageability* and *meaningfulness* of their personal present and future. In this sense, the acquired strategic dimensions form the core of the entrepreneurial behaviour that accompanies resilient processes. It is even possible to define an entrepreneurial profile of the resilient person configured by this set of strategic dimensions linked to entrepreneurial behaviour.

## 5. Towards a Model of Entrepreneurial Resilience

Based on the salutogenic paradigm, we composed some guidelines that outline a comprehensive model of entrepreneurial resilience. From the participants’ narratives, we observed how severe stressful situations have a high probability of having a negative impact on physical and mental well-being. Next, we describe the constituent elements of a comprehensive model of how entrepreneurial resilience is configured in order to face adversity and build life projects. The first block would be the moment of impact of NLEs and would be made up of behavioural, emotional, cognitive and social support processes. The second block would be constituted by educational improvement in which the subjects would learn the set of strategic dimensions of entrepreneurial behaviour. The third block would be characterised by entrepreneurial personal growth through the implementation of the strategic dimensions, facilitating the construction of life projects.

This structure would have a dynamic, integrative and progressive character that would be characterised by the evolution of the subject over a temporal continuum from the appearance of the NLEs to the future projection, passing through educational improvement. At each of these moments, interpersonal and exopersonal GRRs would be identified, formed and used. This model would reflect how the use of the intrapersonal and exopersonal resources by the participants influences the consolidation of a SOC, which, in turn, influences the use of the GRRs. Underlying this structure is the approach to the narrative construction of identity, through autobiographical mnemonic processes (memories and reasoning) that facilitate the generation of a temporal self-awareness over time, as well as a coherent sense of identity.

## 6. Limitations and Future Research

In this research, convenience sampling was selected. RS-14 was used for the selection of the participants and only the data were analysed descriptively. The participants were selected exclusively from the Faculty of Education. Consequently, the study has limitations in terms of generalisability. However, the study was primarily carried out using a phenomenological and interpretative approach. The small number of cases selected and the qualitative nature of the data analysed facilitated an in-depth understanding of what the strategic dimensions of entrepreneurial behaviour are and how entrepreneurial resilience is formed in university students. The study explored a possible entrepreneurial profile in resilient university students but did not explore the contextual GRRs that they have within the university context. In this respect, it is necessary to highlight the lack of quantitative instruments concerning entrepreneurial resilience. Perhaps the strategic dimensions of entrepreneurial behaviour described in the study would constitute possible factors for future psychometric tests related to the subject. Furthermore, the concept of GRRs could be revised, understanding it not only as a set of personal, social or contextual resources that minimise the stressors of everyday life, facilitating adaptation and coping in the face of adverse life circumstances [38], but also as the possibility of generating and implementing strategic dimensions that promote entrepreneurial behaviours that favour the construction of life projects. This reconceptualisation of the GRRs, incorporating the strategic dimensions of entrepreneurial behaviour could take the form of a model of entrepreneurial resilience in the university population.

## 7. Conclusions

This research was developed by taking into account the salutogenic model, with the aim of finding out the strategic entrepreneurial dimensions that are part of resilient processes and understanding how entrepreneurial resilience is formed from the point of view of university students. To this end, the strategic entrepreneurial dimensions and their contribution to the personal projects of resilient university students were identified. The findings of the study contribute firstly to the design of quantitative instruments on the profile of the resilient entrepreneur; second, to the reconceptualisation of the GRRs by introducing the component of strategic dimensions of entrepreneurial behaviour; and finally, to the design and concretisation of the model of entrepreneurial resilience in emerging adulthood.

## Figures and Tables

**Table 1:** Socio-demographic characteristics of the participants (*n* = 276).

Characteristics	Nº of Participants (*n*)	Percentage (%)
Age, m.	21	23.6
18–45 years old	276	100
Gender		
Male	34	12.13
Female	242	87.7
Level of education at university		
First year	151	54.7
Second year	39	14.1
Fourth year	86	31.2
Grade		
Primary Education	114	41.3
Pedagogy	162	58.7

**Table 2:** Participants’ responses related to resilience level.

Resilience Level	Degree of Agreement *n* (%) 1 *=* Totally Disagree 7 = Totally Agree							
	1	2	3	4	5	6	7	Average
1. I generally take things in my stride	10 (3.6)	44 (15.9)	55 (19.9)	63 (22.8)	46 (16.7)	39 (14.1)	19 (6.9)	4.03
2. I am not afraid of difficulties because I have experienced them in the past	9 (3.3)	24 (8.7)	35 (12.7)	63 (22.8)	61 (22.1)	57 (20.7)	27 (9.8)	4.53
3. I am a person with adequate self-esteem	3 (1.1)	21 (7.6)	31 (11.2)	64 (23.2)	78 (28.3)	56 (20.3)	23 (8.3)	4.64
4. I feel I can handle many situations at once	1 (0.4)	10 (3.6)	28 (10.1)	61 (22.1)	97 (35.1)	61 (22.1)	18 (6.5)	4.8
5. I am resolute and determined	1 (0.4)	13 (4.7)	32 (11.6)	55 (19.9)	86 (31.2)	59 (21.4)	30 (10.9)	4.84
6. Self-confidence helps me through difficult times	1 (0.4)	15 (5.4)	36 (13.0)	48 (17.4)	77 (27.9)	57 (20.7)	42 (15.2)	4.9
7. When I am in a difficult situation, I can usually find a way out	0 (0)	2 (0.7)	5 (1.8)	33 (12.0)	68 (24.6)	128 (46.4)	40 (14.5)	5.58
8. I am a disciplined person	1 (0.4)	2 (0.7)	11 (4.0)	24 (8.7)	67 (24.3)	105 (38.0)	66 (23.9)	5.66
9. I usually manage in one way or another	0 (0)	1 (0.4)	6 (2.2)	20 (7.2)	68 (24.6)	113 (40.9)	68 (24.6)	5.78
10. I take an interest in things	0 (0)	2 (0.7)	3 (1.1)	16 (5.8)	60 (21.7)	99 (35.9)	96 (34.8)	5.95
11. My life has meaning	1 (0.4)	1 (0.4)	1 (0.4)	13 (4.7)	53 (19.2)	90 (32.6)	117 (42.4)	6.09
12. I can usually find something to laugh about	0 (0)	1 (0.4)	4 (1.4)	12 (4.3)	56 (20.3)	79 (28.6)	124 (44.9)	6.1
13. I am proud of the things I have achieved	0 (0)	1 (0.4)	0 (0)	8 (2.9)	38 (13.8)	97 (35.1)	132 (47.8)	6.27
14. In an emergency, I am someone people can trust	0 (0)	0 (0)	2 (0.7)	8 (2.9)	32 (11.6)	99 (35.9)	135 (48.9)	6.29

**Table 3:** Distribution of participants according to level of resilience.

Resilience Level	Points in the Scale	*n* (%)
Very low	50–59	0 (0)
Low	60–69	0 (0)
Normal	70–79	25 (9.1)
High	80–89	178 (64.5)
Very high	90–99	73 (26.4)

**Table 4:** Characteristics of the participants (*n* = 10).

Subject	Score RS-14	Sex	Age	Undergraduate Degrees	Course	NLEs
S1P98E37SM	98	Female	37	Pedagogy	Fourth	12
S2P94E25SM	94	Female	25	Pedagogy	Fourth	12
S3P93E32SV	93	Male	32	Pedagogy	Fourth	17
S4P92E26SV	92	Male	26	Primary Education	Second	12
S5P92E24SM	92	Female	24	Pedagogy	Fourth	14
S6P92E23SV	92	Male	23	Pedagogy	First	13 & 17
S7P91E25SM	91	Female	25	Pedagogy	Fourth	13 & 17
S8P91E22SM	91	Female	22	Pedagogy	First	13 & 17
S9P90E30SM	91	Female	24	Primary Education	First	15
S10P90E21SM	90	Female	21	Primary Education	First	8

Note 1: S = subject, P = score on the RS-14 scale, E = age and Sex = male or female. Note 2: items identified in the LEC 5 [62]: 8 = sexual assault, 12 = life-threatening injury or illness, 13 = severe human suffering, 15 = sudden accidental death, 17 = any traumatic event (participants have indicated broken family and poverty).

**Table 5:** Themes and sub-themes.

Themes	Sub-Themes		Codes
Resilience capacity building processes	Intrapersonal	Cognitive processes	Perception Evaluation Adaptation Control Flexibility
		Emotional processes	Knowledge Regulation Expression
		Conductual processes	Approach Avoidance Re-evaluation Reinterpretation Acceptation
	Exopersonal	Social support processes	Explicit Implicit
Strategic entrepreneurial dimensions from the NLEs	Intrapersonal	Basic level	Innovation or creativity Proactivity or initiative Risk-taking
		Specific level	Autonomy Perseverance Problem solving Self-confidence Achievement orientation
Future projection processes of the self	Intrapersonal	Personal objectives	Academic Welfare Labour Relational
		Personal efforts	Academic Welfare Labour Relational
		Personal projects	Restructuring Typology Evaluation Ranking
	Exopersonal	Contextual variables	Economic Material

## Data Availability

Not applicable.
